# Predictors of outcome after catheter ablation for atrial fibrillation: Group analysis categorized by age and type of atrial fibrillation

**DOI:** 10.1111/anec.13020

**Published:** 2022-12-16

**Authors:** Tetsuya Uemura, Hidekazu Kondo, Hiroki Sato, Masaki Takahashi, Tetsuji Shinohara, Kazuki Mitarai, Akira Fukui, Kei Hirota, Tomoko Fukuda, Nozomi Kodama, Miho Miyoshi, Naoko Ogawa, Masato Wada, Hirochika Yamasaki, Kenzo Iwanaga, Akihiro Uno, Katsunori Tawara, Keisuke Yonezu, Hidefumi Akioka, Yasushi Teshima, Kunio Yufu, Mikiko Nakagawa, Naohiko Takahashi

**Affiliations:** ^1^ Department of Cardiology and Clinical Examination, Faculty of Medicine Oita University Yufu Oita Japan

**Keywords:** age, catheter ablation, paroxysmal atrial fibrillation, persistent atrial fibrillation, recurrence

## Abstract

**Background:**

The outcome of catheter ablation could probably differ among patients with atrial fibrillation (AF), depending on age and AF type. We aimed to investigate the difference in predictors of outcome after catheter ablation for AF among the patient categories divided by age and AF type.

**Methods and Results:**

A total of 396 patients with AF (mean age 65.69 ± 11.05 years, 111 women [28.0%]) who underwent catheter ablation from January 2018 to December 2019 were retrospectively analyzed. We divided the patients into four categories: patients with paroxysmal AF (PAF) or persistent AF (PeAF) who were 75 years or younger (≤75 years) or older than 75 years (>75 years). Kaplan–Meier survival analysis demonstrated that patients with PAF aged ≤75 years had the lowest AF recurrence among the four groups (log‐rank test, *p* = .0103). In the patients with PAF aged ≤75 years (*N* = 186, 46.7%), significant factors associated with recurrence were female sex (*p* = .008) and diabetes (*p* = .042). In the patients with PeAF aged ≤75 years (*N* = 142, 35.9%), the only significant factor associated with no recurrence was medication with a renin‐angiotensin system inhibitor (*p* = .044). In the patients with PAF aged >75 years (*N* = 53, 14.4%), diabetes was significantly associated with AF recurrence (*p* = .021). No significant parameters were found in the patients with PeAF aged >75 years (*N* = 15, 4.1%).

**Conclusions:**

Our findings indicate that the risk factors for AF recurrence after catheter ablation differed by age and AF type.

## Graphical abstract

The patients with atrial fibrillation (AF) divided by age and the type AF showed significant difference of AF recurrence after catheter ablation. The significant factors associated with AF recurrence were found in each category.

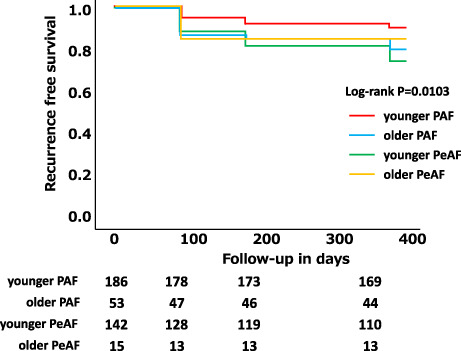

## 1 INTRODUCTION

Catheter ablation for atrial fibrillation (AF), especially pulmonary vein antrum isolation (PVAI), is effective for maintaining sinus rhythm; however, the efficacy is limited by the type of AF (paroxysmal or persistent) (Bhargava et al., 2009). The ability for catheter ablation to maintain sinus rhythm is greater in patients with paroxysmal AF (PAF) than in those with persistent AF (PeAF) (Parkash et al., 2010; Brooks et al., 2010). This could be due to progression of AF substrates out of the pulmonary vein (PV) and the presence of non‐PV targets that remain after PVAI (Terricabras et al., 2020).

The impact of age on the outcomes after AF ablation is controversial. In studies that investigated the outcomes of AF catheter ablation focusing on differences in age, no significant variations in the overall success rate by age were noted (Natale et al., 2021; Bunch et al., 2010; Bahnson et al., 2022). In contrast, a small study that included patients with persistent AF undergoing cryoballoon catheter ablation showed that the older group (>75 years) achieved a lower success rate (36.1%) than the younger group (≤75 years) (47.0%) (Vermeersch et al., 2021).

There are several different predictors for AF recurrence after catheter ablation between patients with PAF and PeAF or younger and older patients (Bhargava et al., 2009; Buiatti et al., 2016; Fujino et al., 2020). The purpose of this study was to investigate the difference in predictors of outcome after catheter ablation for AF among patient categories divided by age and AF type.

## 2 MATERIALS AND METHODS

The data supporting our findings of this study are available from the corresponding author upon reasonable request.

### 2.1 Patient selection

The retrospective study enrolled 396 patients with PAF (*N* = 239) and PeAF (*N* = 157) who underwent radiofrequency catheter ablation for AF at our institute between January 2018 and December 2019. Their mean age was 65.69 ± 11.05 years. The study group comprised 111 females and 285 males. Sixty‐eight patients were aged 76 years and older (labeled as “older”) and 328 were aged 75 years and younger (“younger”).

Patients with prior AF ablation, cardiovascular implantable electronic devices, cardiopulmonary disease, or structural heart disease were excluded from the study. PAF and PeAF were defined according to the 2017 HRS/EHRA/ECAS/APHRS/SOLAECE expert consensus statement (Calkins et al., 2018). Briefly, PAF is defined as an episode of AF that terminates spontaneously or with intervention in <7 days, and PeAF is defined as episodes that are sustained for >7 days and are not self‐terminating. Transthoracic and transesophageal echocardiograms were performed using the Vivid 7 ultrasound system (GE Vingmed) before ablation to evaluate the left ventricular function and left atrial diameter (LAD) and to exclude the presence of thrombi.

Experienced physicians recorded the medical history, medication regimes, and body mass index of all patients. All patients underwent physical examination, ECG, and blood testing (including renal function, Hb, HbA1c, NT‐proBNP, and C‐reactive protein).

### 2.2 Follow‐up

Follow‐up was performed at 1, 3, 6, and 12 months after catheter ablation using a 12‐lead electrocardiogram and 24‐h Holter monitoring at each visit. Any atrial tachyarrhythmia lasting ≥1 min was considered a recurrence. In addition to palpitation, patients were asked to check whether their pulse was regular in their free time. If recurrence was suspected, additional 24‐h Holter monitoring was performed. The discontinuation of antiarrhythmic drugs was recommended at the 3‐month follow‐up.

### 2.3 Pulmonary vein antrum isolation by catheter ablation

Contact force‐guided PVAI was performed by two operators. Circumferential PVAI was performed with integrated 3D images using the open‐irrigated ThermoCool SmartTouch catheter (Biosense Webster). The ablation catheter was advanced into the left atrium (LA) using a long sheath. Radiofrequency energy was delivered at 30 W in the anterior aspect of the circumferential PVAI line and at 25 W in the posterior aspect using the Stockert 70 generator system (Biosense Webster) radiofrequency generator. The operator attempted to maintain a contact force between 10 and 20 g during PVAI. While radiofrequency energy was being delivered, the catheter tip was dragged by approximately 2 mm every 5–15 s. The endpoint of PVAI was the elimination of all PV potentials recorded by a circular catheter (Lasso, Biosense Webster) placed at the ostium of the PV and the PV‐to‐LA block during pacing from 10 pairs of the circular catheter at 10 V output with 1‐ms pulse width. Isoproterenol (4 μg) was injected intravenously to induce AF in the non‐PV foci. When a non‐PV focus was identified, focal ablation was performed at the foci, except for one in the superior vena cava (SVC) where segmental isolation was performed. SVC isolation was performed if the length of the SVC sleeve was regarded >30 mm (Higuchi et al., 2010). Cavotricuspid isthmus (CTI) linear ablation was also performed if atrial flutter was documented before ablation or induced during the ablation procedure.

### 2.4 Statistical analysis

Baseline clinical characteristics are presented as mean with standard deviation or frequency with percentage, as appropriate. For continuous variables, normality of the distribution was tested using Shapiro–Wilk test. For continuous variables, an unpaired t‐test was used to test a difference between the PAF and PeAF groups. For categorical variables, chi‐square test and Fisher's exact test were used. A value of *p* < .05 was considered significant. Potential risk factors for AF recurrence were investigated using univariate logistic regression. All computations were performed using the SPSS statistical software (version 26.0) running on Windows 10 (Microsoft).

## 3 RESULTS

### 3.1 Patient characteristics

The characteristics of patients with AF in this study are shown in Table 1. This study enrolled a total of 396 patients (mean age 65.69 ± 11.05 years; 111 females). Of the 396 patients, 157 (39.6%) had PeAF, and 68 were aged >75 years, with an average age of 78.9 ± 2.9 years. The average age of patients aged ≤75 years was 62.9 ± 10.1 years (*p* < .001). All patients underwent PVAI, and 208 patients (52.5%) underwent isolation of the SVC. There were no significant differences in the execution rate of SVC isolation among the four groups. Based on their age and type of AF, patients were classified into group 1: ≤75 years and PAF (labeled as “younger PAF,” *n* = 186), group 2: ≤75 years and PeAF (“younger PeAF,” *n* = 142), group 3: >75 years and PAF (“older PAF,” *n* = 53), and group 4: >75 years and PeAF (“older PeAF,” *n* = 15). The basic demographics of the four groups are listed in Table S1. There were significant differences in the number of male patients, creatinine clearance, plasma NT‐proBNP level, height, body weight, the mean CHADS2 score, amiodarone medication, antiarrhythmic medication, LAD, left ventricular ejection fraction, and E/e′ among the four groups. In contrast, there were no significant differences in the prevalence of hypertension, diabetes, serum creatinine levels, HbA1c, C‐reactive protein, BMI, treatment with a renin‐angiotensin system (RAS) inhibitor, and β‐blocker use among the groups.

**TABLE 1:** Baseline clinical characteristics of patients enrolled in this study (*n* = 396)

Male sex, *n* (%)	285 (72.0)
Age—years	65.7 ± 11.1
Type of AF	
Paroxysmal, *n* (%)	239 (60.4)
Persistent, *n* (%)	157 (39.6)
Stroke, *n* (%)	34 (8.6)
Hypertension, *n* (%)	238 (60.1)
Diabetes, *n* (%)	61 (15.4)
Laboratory data	
Creatinine—mg/dl	0.91 ± 0.45
Creatinine clearance—ml/min	79.6 ± 31.3
Median NT‐proBNP (IQR)—pg/ml	267 (98–604)
HbA1c—%	5.87 ± 0.81
C‐reactive protein—mg/dl	0.19 ± 0.59
Height—m	1.64 ± 0.09
Weight—kg	67.2 ± 13.8
Body mass index—kg/m^2^	24.8 ± 4.04
CHADS2	
0, *n* (%)	104 (26.3)
1, *n* (%)	152 (38.4)
2, *n* (%)	98 (24.7)
3, *n* (%)	30 (7.6)
4, *n* (%)	11 (2.8)
5, *n* (%)	1 (0.2)
Medication	
ACEI/ARB, *n* (%)	162 (40.9)
Beta‐blocker, *n* (%)	174 (43.9)
Amiodarone, *n* (%)	51 (12.9)
Antiarrhythmic, *n* (%)	72 (18.2)
Measurements by echocardiogram	
Left atrial diameter—mm	40.3 ± 0.5
Left ventricular ejection fraction—%	63.3 ± 10.2
E/e′	11.4 ± 4.5

*Note*: Plus‐minus values are means ± SD.

Abbreviation: IQR, interquartile range.

### 3.2 Kaplan–Meier MACCE‐free estimation

**FIGURE 1:**
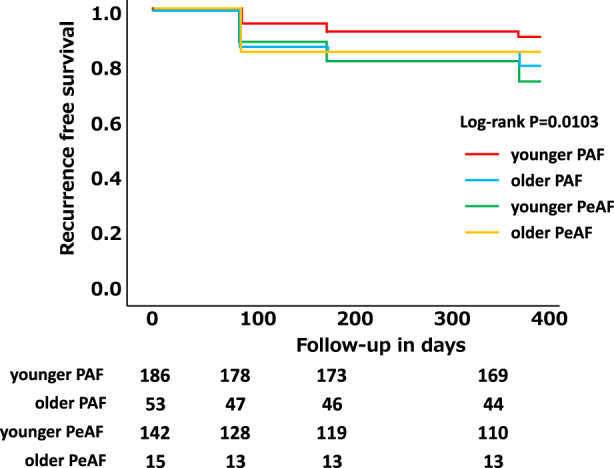
Kaplan–Meier curves showing AF recurrence free survival among four groups. There is a significant difference among four groups (log‐rank *p* = .0103).

Kaplan**–**Meier survival analysis revealed that the AF recurrence rate was significantly different among the four groups at the 12‐month follow‐up (log‐rank *p* = .0103, Figure 1). The AF‐free survival rate of the younger PAF group was the highest, whereas that of the younger PeAF group was the lowest among the four groups.

### 3.3 Predictor of AF recurrence in the younger PAF group, aged ≤75 years

The baseline clinical, echocardiographic, and biochemical characteristics of patients with or without AF recurrence are shown in Table 2. The category of patients with PAF aged ≤75 years included 186 patients (72.6% males). Univariate analysis revealed that the significant factors associated with recurrence were female sex (*p* = .008) and diabetes (*p* = .042). In addition, multivariate analysis revealed that female sex (*p* = .005) and the prevalence of diabetes (*p* = .019) were independent predictive factors for AF recurrence.

**TABLE 2:** Predictor of AF recurrence in the younger PAF group

	AF recurrence (−)	AF recurrence (+)	*p* value	Multivariate analysis		
	(*n* = 169)	(*n* = 17)		Odds ratio	95% CI	*p* value
Male sex, *n* (%)	128 (75.7)	7 (41.2)	.008	0.21	0.070–0.622	.005
Age—years	63.8 ± 10.2	66.2 ± 8.4	.335	1.03	0.91–1.03	.417
Stroke, *n* (%)	15 (8.9)	1 (5.9)	1.000			
Hypertension, *n* (%)	99 (58.6)	9 (52.9)	.797			
Diabetes, *n* (%)	18 (10.7)	5 (29.4)	.042	4.54	1.219–16.667	.019
Creatinine—mg/dl	0.9 ± 0.2	0.8 ± 0.2	.275			
Creatinine clearance—ml/min	85.0 ± 32.8	80.5 ± 27.4	.592			
Median NT‐proBNP (IQR)—pg/ml	121 (57–269)	182 (84–543)	.650			
HbA1c—%	5.8 ± 1.0	6.0 ± 0.7	.357			
C‐reactive protein—mg/dl	0.3 ± 0.4	0.2 ± 0.3	.730			
Height—m	1.6 ± 0.1	1.6 ± 0.1	.446			
Weight—kg	67.2 ± 13.1	64.7 ± 12.1	.456			
Body mass index—kg/m^2^	24.7 ± 3.9	24.5 ± 3.2	.858			
CHADS2						
0, *n* (%)	59 (34.9)	5 (0.0)	.950			
1, *n* (%)	70 (41.4)	8 (47.1)				
2, *n* (%)	25 (14.8)	3 (17.6)				
3, *n* (%)	12 (7.1)	1 (5.9)				
4, *n* (%)	3 (1.8)	0 (0.0)				
5, *n* (%)	0 (0.0)	0 (0.0)				
Medication						
ACEI/ARB, *n* (%)	62 (36.7)	7 (41.2)	.794			
Beta‐blocker, *n* (%)	72 (42.6)	6 (35.3)	.616			
Amiodarone, *n* (%)	14 (8.3)	2 (11.8)	.644			
Antiarrhythmic, *n* (%)	42 (24.9)	6 (35.3)	.386			
Echocardiographic parameter						
Left atrial diameter—mm	38.5 ± 5.6	38.2 ± 3.9	.811			
Left ventricular ejection fraction—%	65.4 ± 8.8	66.8 ± 5.7	.499			
E/e′	10.9 ± 4.3	11.3 ± 4.9	.706			

*Note*: Plus‐minus values are means ± SD.

Abbreviation: IQR, interquartile range.

### 3.4 Predictor of AF recurrence in the older PAF group, aged >75 years

The baseline clinical, echocardiographic, and biochemical characteristics of patients with or without AF recurrence are shown in Table 3. The group included 53 patients (45.3% males). Univariate analysis revealed that the only significant factor associated with recurrence was diabetes (*p* = .021), which was also confirmed as an independent predictive factor for AF recurrence in multivariate analysis (*p* = .010).

**TABLE 3:** Predictor of AF recurrence in the older PAF group

	AF recurrence (−)	AF recurrence (+)	*p* value	Multivariate analysis		
	(*n* = 44)	(*n* = 9)		Odds ratio	95% CI	*p* value
Male sex, *n* (%)	21 (47.7)	3 (33.3)	.488	0.75	0.113–4.348	.752
Age—years	79.0 ± 2.7	79.4 ± 3.5	.656	1.15	0.833–1.587	.377
Stroke, *n* (%)	8 (0.2)	0 (0)	.324			
Hypertension, *n* (%)	31 (70.5)	6 (66.7)	1.000			
Diabetes, *n* (%)	4 (9.1)	4 (44.4)	.021	16.6	1.219–16.667	.010
Creatinine—mg/dL	0.9 ± 0.4	0.9 ± 0.2	.964			
Creatinine clearance—ml/min	55.8 ± 16.9	52.0 ± 16.6	.541			
Median NT‐proBNP (IQR)—pg/ml	251 (156–433)	538 (345–794)	.276			
HbA1c—%	5.9 ± 0.5	6.1 ± 0.5	.266			
C‐reactive protein—mg/dl	0.1 ± 0.2	0.1 ± 0.1	.205			
Height—m	1.6 ± 0.1	1.6 ± 0.1	.749			
Weight—kg	58.9 ± 10.3	58.4 ± 13.8	.913			
Body mass index—g/m^2^	24.0 ± 3.2	24.1 ± 4.6	.930			
CHADS2						
0, *n* (%)	0 (0)	0 (0)	.119			
1, *n* (%)	6 (13.6)	2 (22.2)				
2, *n* (%)	27 (61.4)	2 (22.2)				
3, *n* (%)	7 (15.9)	4 (44.4)				
4, *n* (%)	3 (6.8)	1 (11.1)				
5, *n* (%)	1 (2.3)	0 (0.0)				
Medication						
ACEI/ARB, *n* (%)	20 (45.5)	4 (44.4)	1.000			
Beta‐blocker, *n* (%)	17 (38.6)	4 (44.4)	1.000			
Amiodarone, *n* (%)	1 (2.3)	1 (11.1)	.314			
Antiarrhythmic, *n* (%)	5 (11.4)	1 (11.1)	1.000			
Echocardiographic parameter						
Left atrial diameter—mm	39.6 ± 5.8	39.6 ± 4.8	.987			
Left ventricular ejection fraction—%	65.6 ± 10.2	65.7 ± 8.2	.972			
E/e′	14.6 ± 6.1	15.8 ± 5.2	.584			

*Note*: Plus‐minus values are means ± SD.

Abbreviation: IQR, interquartile range.

### 3.5 Predictor of AF recurrence in the younger PeAF group, aged ≤75 years

The baseline clinical, echocardiographic, and biochemical characteristics of patients with or without AF recurrence are shown in Table 4. The group included 142 patients (83.1% males). No independent factors predicting AF recurrence were identified by multivariate analysis. The only significant factor associated with no recurrence was medication with RAS inhibitors (*p* = .044).

**TABLE 4:** Predictor of AF recurrence in the younger PeAF group

	AF recurrence (−)	AF recurrence (+)	*p* value	Multivariate analysis		
	(*n* = 110)	(*n* = 32)		Odds ratio	95% CI	*p* value
Male sex, *n* (%)	91 (82.7)	27 (84.4)	1.000	1.03	0.313–3.012	.964
Age—years	61.7 ± 10.0	61.2 ± 10.1	.812	0.99	0.95–1.04	.788
Stroke, *n* (%)	6 (5.5)	2 (6.3)	1.000			
Hypertension, *n* (%)	67 (60.9)	14 (43.8)	.105			
Diabetes, *n* (%)	23 (20.1)	5 (15.6)	.619			
Creatinine—mg/dl	1.0 ± 0.7	1.0 ± 0.4	.901			
Creatinine clearance—ml/min	85.5 ± 30.5	83.2 ± 27.0	.712			
Median NT‐proBNP (IQR)—pg/ml	530 (277–902)	478 (237–663)	.335			
HbA1c—%	6.0 ± 0.6	5.8 ± 0.5	.360			
C‐reactive protein—mg/dl	0.2 ± 0.5	0.2 ± 0.5	.947			
Height—m	1.7 ± 0.1	1.7 ± 0.1	0.839			
Weight—kg	71.6 ± 14.9	70.7 ± 12.1	.770			
Body mass index—kg/m^2^	25.6 ± 4.7	25,2 ± 3.6	.649			
CHADS2						
0, *n* (%)	28 (25.5)	11 (34.4)	.409			
1, *n* (%)	49 (44.5)	15 (46.9)				
2, *n* (%)	28 (25.5)	5 (15.6)				
3, *n* (%)	4 (3.5)	0 (0.0)				
4, *n* (%)	1 (1.0)	1 (3.1)				
5, *n* (%)	0 (0.0)	0 (0.0)				
Medication						
ACEI/ARB, *n* (%)	53 (48.2)	9 (28.1)	.044	0.42	0.164–1.010	.059
Beta‐blocker, *n* (%)	54 (49.1)	11 (34.4)	.162			
Amiodarone, *n* (%)	25 (22.7)	7 (21.9)	1.000			
Antiarrhythmic, *n* (%)	12 (10.9)	5 (15.6)	.537			
Echocardiographic parameter						
Left atrial diameter—mm	42.2 ± 5.1	43.8 ± 4.7	.129			
Left ventricular ejection fraction—%	58.8 ± 11.2	62.4 ± 8.9	.102			
E/e′	10.5 ± 3.6	10.6 ± 3.5	.864			

*Note*: Plus‐minus values are means ± SD.

Abbreviation: IQR, interquartile range.

### 3.6 Predictor of AF recurrence in the older PeAF group, aged >75 years

The baseline clinical, echocardiographic, and biochemical characteristics of patients with or without AF recurrence are shown in Table S2. The group included 15 patients (53.3% males), and no significant parameters were found.

### 3.7 Predictor of AF recurrence in the PeAF group regardless of age

The group of patients with persistent AF is small and with low number of events, therefore, we combined the two PeAF groups regardless of age. The basic demographics of the three groups (PAF ≤75, PAF >75 and PeAF) are listed in Table S3. Kaplan**–**Meier survival analysis revealed that the AF recurrence rate was significantly different among the three groups at the 12‐month follow‐up (log‐rank *p* = .0053, Figure S1). The PeAF group included 157 patients. The baseline clinical, echocardiographic, and biochemical characteristics of all PeAF patients with or without AF recurrence are shown in Table S4. Univariate analysis revealed that the factor associated with recurrence was medication with RAS inhibitors (*p* = .078), but it was not significant. No independent factors predicting AF recurrence were identified by multivariate analysis, even if we included medication with RAS inhibitors (*p* = .059).

## 4 DISCUSSION

The main findings of this study are as follows: (1) the clinical success rates of AF ablation after 1 year were significantly different among the four groups categorized by age and AF type; (2) the AF‐free survival of the younger PAF group was the greatest, whereas that of the younger PeAF group was the lowest among the groups; (3) univariate analysis to predict AF recurrence in each group revealed that the prevalence of diabetes was significantly associated with AF recurrence in the younger and older PAF groups, whereas female sex was a significant predictor in the younger PAF group. In addition, medication with RAS inhibitors was significantly associated with no recurrence in the younger PeAF group; and (4) multivariate analysis revealed that the prevalence of diabetes and female sex were independent predictors of AF recurrence in the younger PAF group, whereas only the prevalence of diabetes was an independent predictor in the older PAF group.

Consistent with the results of our study, previous studies have shown that female sex and the presence of diabetes are independent predictors of AF recurrence after catheter ablation (Wang et al., 2020; Creta et al., 2019; Arora et al., 2018). Female sex has been reported to be associated with the presence of left atrial low‐voltage areas (Huo et al., 2018). In addition, Takigawa et al. reported that the prevalence of non‐PV triggers was significantly higher in women than in men (16% vs. 8.4%) (Takigawa et al., 2013). Another study reported that parasympathetic nervous activity, potentially affecting PAF vulnerability (Chen et al., 2014), is significantly enhanced in women than in men before and after AF ablation (Yu et al., 2018). The presence of these arrhythmogenic factors may contribute to higher AF recurrence rates in the younger PAF group. Sex‐related differences in parasympathetic regulation diminish with age (Kuo et al., 1999), which could be a reason why sex differences were detected only in the younger PAF group.

Diabetes is known to promote atrial remodeling associated with AF recurrence (Wang et al., 2019). In our study, diabetes was associated with recurrence in patients with PAF regardless of age but not associated in patients with PeAF. As described previously, the cardiac autonomic nervous system more greatly contributes to the pathogenesis of PAF than to that of PeAF. Based on these findings, diabetes might enhance AF vulnerability by deteriorating the cardiac autonomic nervous function.

Interestingly, treatment with a RAS inhibitor was significantly associated with no recurrence of AF only in the younger PeAF group in this study. The cardioprotective effects of RAS inhibitors have been widely accepted. RAS inhibitors attenuate cardiac remodeling by suppressing atrial inflammation and fibrosis (Schieffer et al., 1994; Nunez et al., 1997; Zhu et al., 1997). However, the preventive effect of RAS inhibitors on AF recurrence after catheter ablation remains controversial. Previous studies have suggested that RAS inhibitors are effective for the prevention of AF recurrence after radiofrequency catheter ablation (Wang et al., 2016; Cui et al., 2015; Tian et al., 2019), while other studies have reported that RAS inhibitors have no preventive effect (Tayebjee et al., 2010; Patel et al., 2010). Atrial remodeling progresses depending on age. Our study may provide the potential efficacy of RAS inhibitor therapy in the younger patient category before remodeling develops with advancing age. The PeAF‐limited effect of RAS inhibitors might be explained by the fact that the causes of PAF are often multifactorial (parasympathetic nervous activity, etc.) compared to those of PeAF, which are mainly considered to be atrial substrates. RAS inhibitors might not be able to affect factors such as increased parasympathetic nervous activity, resulting in no predictive value in patients with PAF. This study has some limitations. First, we followed up the patients for only 1 year. This period might be too short to monitor for AF recurrence. Second, the patients were not assessed for recurrent AF using an insertable cardiac monitor. Therefore, we might have missed AF recurrence in asymptomatic patients. Third, this study was not a multicenter study and the number of patients was relatively small. Fourth, some results of this study are different from previous reports, but it seems to be related to study population. In conclusion, our findings suggest that the predictors for AF recurrence after radiofrequency catheter ablation should be considered depending on age and AF type. Appropriate management for diabetes would improve the success rate of catheter ablation for PAF irrespective of age. In addition, RAS inhibitors might play a favorable role in efficacy of catheter ablation in younger PeAF group. However, due to limitations of the study, further follow‐up and a multicenter study are needed to validate the consistency and reproducibility of the results.

## 5 AUTHOR CONTRIBUTIONS

Tetsuya Uemura and Hidekazu Kondo wrote the draft of this article and designed the study. Hiroki Sato helped to perform correct statistical analysis. Masaki Takahashi, Tetsuji Shinohara, Kazuki Mitarai, Akira Fukui, and Kei Hirota performed catheter ablation and followed‐up the patients. Tomoko Fukuda, Nozomi Kodama, Miho Miyoshi, Naoko Ogawa, Masato Wada, Hirochika Yamasaki, Kenzo Iwanaga, Akihiro Uno, Katsunori Tawara, Keisuke Yonezu, Hidefumi Akioka, Yasushi Teshima, Kunio Yufu, and Mikiko Nakagawa gave the advice in terms of interpretation of the data and planning the clinical research. Naohiko Takahashi made the decision regarding final approval of this article.

## FUNDING INFORMATION

None.

## CONFLICT OF INTEREST

The authors declared no conflict of interest for this article.

## COMPLIANCE WITH ETHICAL STANDARDS

The study was conducted in accordance with the ethics review board of Oita University. Informed consent was obtained from all subjects.

## CLINICAL TRIAL REGISTRATION

This study does not meet the definition of clinical trial. This study is a retrospective study and is not classified as an interventional trial.

## 6 ETHICS STATEMENT

This retrospective study was conducted in accordance with the ethical standards of the institutional and national research committee and with the 1964 Helsinki Declaration and its later amendments or comparable ethical standards. The Institutional Review Board (IRB) of Oita University approved this study. Informed consent was obtained from all patients by the opt‐out method.

## Supporting information


Figure S1

Table S1

Table S2

Table S3

Table S4

## Data Availability

The data supporting our findings of this study are available from the corresponding author upon reasonable request.
