# Supplementary material for: Predictors of outcome after catheter ablation for atrial fibrillation: Group analysis categorized by age and type of atrial fibrillation
Source: Ann Noninvasive Electrocardiol. 2022 Dec 16;28(2):e13020. doi: 10.1111/anec.13020 (PMC10023880; doi:10.1111/anec.13020)
Supplement: Supplementary file 1 — Figure S1 [file ANEC-28-e13020-s002.pptx]

## Slide 1
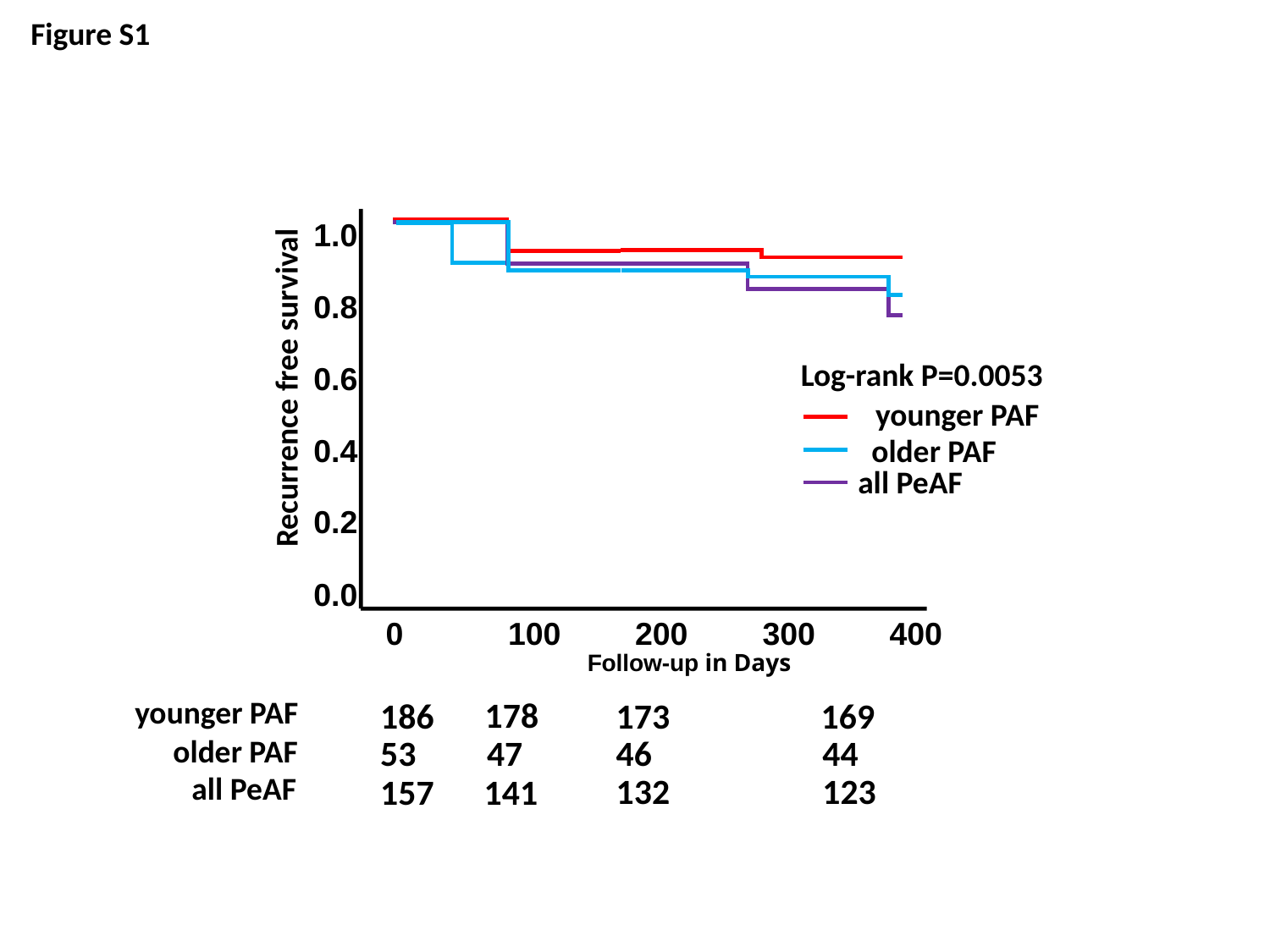

Figure S1
1.0
0.8
Log-rank P=0.0053
0.6
Recurrence free survival
younger PAF
older PAF
0.4
all PeAF
0.2
0.0
0
100
200
300
400
Follow-up in Days
younger PAF
178
186
173
169
older PAF
53
46
44
47
all PeAF
132
123
141
157
