# Supplementary material for: Predictors of outcome after catheter ablation for atrial fibrillation: Group analysis categorized by age and type of atrial fibrillation
Source: Ann Noninvasive Electrocardiol. 2022 Dec 16;28(2):e13020. doi: 10.1111/anec.13020 (PMC10023880; doi:10.1111/anec.13020)
Supplement: Supplementary file 2 — Table S1 [file ANEC-28-e13020-s004.docx]

**Table S1. Baseline and procedural characteristics of four groups, younger PAF, younger PeAF, older PAF and older PeAF.**

|  |  | **Younger, PAF** | | | **Younger, PeAF** | | | **Older, PAF** | | | **Older, PeAF** | | | **p value** |
| --- | --- | --- | --- | --- | --- | --- | --- | --- | --- | --- | --- | --- | --- | --- |
|  |  | **(n=186)** | | | **(n=142)** | | | **(n=53)** | | | **(n=15)** | | |  |
| **Male sex, *n* (%)** | | **135** |  | **(72.6)** | **118** |  | **(83.1)** | **24** |  | **(45.3)** | **8** |  | **(53.3)** | **<0.001** |
| **Age – years** | | **64.0** | **±** | **10.1** | **61.6** | **±** | **10.0** | **79.1** | **±** | **2.8** | **78.3** | **±** | **3.2** | **<0.001** |
| **Stroke, *n* (%)** | | **16** |  | **(8.6)** | **8** |  | **(5.6)** | **8** |  | **(15.1)** | **2** |  | **(13.3)** | **0.210** |
| **Hypertension, *n* (%)** | | **108** |  | **(58.1)** | **81** |  | **(57.0)** | **37** |  | **(70.0)** | **12** |  | **(80.0)** | **0.148** |
| **Diabetes, *n* (%)** | | **23** |  | **(12.4)** | **28** |  | **(19.7)** | **8** |  | **(15.1)** | **2** |  | **(13.3)** | **0.341** |
| **Laboratory data** | |  |  |  |  |  |  |  |  |  |  |  |  |  |
|  | **Creatinine – mg/dL** | **0.9** | **±** | **0.2** | **1.0** | **±** | **0.7** | **0.9** | **±** | **0.3** | **0.9** | **±** | **0.3** | **0.085** |
|  | **Creatinine clearance – mL/min** | **84.6** | **±** | **32.3** | **85.0** | **±** | **29.6** | **55.1** | **±** | **16.7** | **54.3** | **±** | **17.0** | **<0.001** |
|  | **Median NT-proBNP (IQR) – pg/mL** | **123** |  | **(57 – 288)** | **528** |  | **(253 – 878)** | **268** |  | **(160 – 481)** | **934** |  | **(774 – 2009)** | **<0.001** |
|  | **HbA1c – %** | **5.8** | **±** | **1.0** | **5.9** | **±** | **0.6** | **5.9** | **±** | **0.5** | **6.0** | **±** | **0.5** | **0.392** |
|  | **C-reactive protein –**  **mg/dL** | **0.3** | **±** | **1.3** | **0.2** | **±** | **0.5** | **0.1** | **±** | **0.2** | **0.2** | **±** | **0.2** | **0.757** |
|  | **Height – m** | **1.6** | **±** | **0.1** | **1.7** | **±** | **0.1** | **1.6** | **±** | **0.1** | **1.6** | **±** | **0.1** | **<0.001** |
|  | **Weight – kg** | **66.9** | **±** | **13.0** | **71.4** | **±** | **14.2** | **58.8** | **±** | **10.8** | **59.5** | **±** | **11.7** | **<0.001** |
|  | **Body mass index –**  **kg/m^2^** | **24.6** | **±** | **3.9** | **25.5** | **±** | **4.4** | **24.0** | **±** | **3.4** | **23.8** | **±** | **3.5** | **0.411** |
| **CHADS2** | |  |  |  |  |  |  |  |  |  |  |  |  |  |
|  | **0, *n* (%)** | **64** |  | **(34.4)** | **39** |  | **(27.5)** | **0** |  | **(0.0)** | **0** |  | **(0.0)** | **<0.001** |
|  | **1, *n* (%)** | **78** |  | **(41.9)** | **64** |  | **(45.1)** | **8** |  | **(15.1)** | **2** |  | **(13.3)** |  |
|  | **2, *n* (%)** | **28** |  | **(15.1)** | **33** |  | **(23.2)** | **29** |  | **(54.7)** | **9** |  | **(60.0)** |  |
|  | **3, *n* (%)** | **13** |  | **(7.0)** | **4** |  | **(2.8)** | **11** |  | **(20.8)** | **2** |  | **(13.3)** |  |
|  | **4, *n* (%)** | **3** |  | **(1.6)** | **2** |  | **(1.4)** | **4** |  | **(7.5)** | **2** |  | **(13.3)** |  |
|  | **5, *n* (%)** | **0** |  | **(0.0)** | **0** |  | **(0.0)** | **1** |  | **(1.9)** | **0** |  | **(0.0)** |  |
| **Medication** | |  |  |  |  |  |  |  |  |  |  |  |  |  |
|  | **ACEI/ARB, *n* (%)** | **69** |  | **(37.1)** | **62** |  | **(43.7)** | **24** |  | **(45.3)** | **7** |  | **(46.7)** | **0.534** |
|  | **Beta-blocker, *n* (%)** | **78** |  | **(41.9)** | **65** |  | **(45.8)** | **21** |  | **(39.6)** | **10** |  | **(66.7)** | **0.257** |
|  | **Amiodarone, *n* (%)** | **16** |  | **(8.6)** | **32** |  | **(22.5)** | **2** |  | **(3.8)** | **1** |  | **(6.7)** | **0.000** |
|  | **Antiarrhythmic, *n* (%)** | **48** |  | **(25.8)** | **17** |  | **(12.0)** | **6** |  | **(11.3)** | **1** |  | **(6.7)** | **0.003** |
| **Measurements by echocardiogram** | |  |  |  |  |  |  |  |  |  |  |  |  |  |
|  | **Left atrial diameter –mm** | **38.5** | **±** | **5.5** | **42.6** | **±** | **5.0** | **39.6** | **±** | **5.6** | **43.9** | **±** | **4.5** | **<0.001** |
|  | **Left ventricular ejection fraction – %** | **65.5** | **±** | **8.5** | **59.6** | **±** | **10.8** | **65.6** | **±** | **9.8** | **62.4** | **±** | **14.6** | **<0.001** |
|  | **E/e'** | **11.0** | **±** | **4.3** | **10.5** | **±** | **3.6** | **14.8** | **±** | **6.0** | **12.8** | **±** | **4.5** | **<0.001** |

Plus-minus values are means ± SD. IQR means interquartile range.
