# Supplementary material for: Predictors of outcome after catheter ablation for atrial fibrillation: Group analysis categorized by age and type of atrial fibrillation
Source: Ann Noninvasive Electrocardiol. 2022 Dec 16;28(2):e13020. doi: 10.1111/anec.13020 (PMC10023880; doi:10.1111/anec.13020)
Supplement: Supplementary file 3 — Table S2 [file ANEC-28-e13020-s001.docx]

**Table S2. Predictor of AF recurrence in the older PeAF group**

|  |  |  |  |  |  | **Multivariate analysis** | | |
| --- | --- | --- | --- | --- | --- | --- | --- | --- |
|  |  | **AF recurrence (-)** |  | **AF recurrence (+)** | **p value** | **Odds ratio** | **95% CI** | **p value** |
|  |  | **(n=13)** |  | **(n=2)** |  |  |  |  |
| **Male sex, *n* (%)** | | **7 (53.8)** |  | **1 (50.0)** | **1.000** | **N.A.** | | |
| **Age – years** | | **78.7 ± 3.3** |  | **76.0 ± 0.0** | **0.999** |  |  |  |
| **Stroke, *n* (%)** | | **2 (15.4)** |  | **0 (0)** | **1.000** |  |  |  |
| **Hypertension, *n* (%)** | | **11 (84.6)** |  | **1 (50.0)** | **0.371** |  |  |  |
| **Diabetes, *n* (%)** | | **2 (15.4)** |  | **0 (0)** | **1.000** |  |  |  |
| **Creatinine – mg/dL** | | **0.9 ± 0.3** |  | **0.8 ± 0.2** | **0.389** |  |  |  |
| **Creatinine clearance – mL/min** | | **53.8 ± 17.9** |  | **57.7 ± 12.7** | **0.775** |  |  |  |
| **Median NT-proBNP (IQR) – pg/mL** | | **934**  **(811 - 1977)** |  | **2071**  **(1404 - 2737)** | **0.348** |  |  |  |
| **HbA1c – %** | | **6.1 ± 0.5** |  | **5.7 ± 0.2** | **0.278** |  |  |  |
| **C-reactive protein – mg/dL** | | **0.2 ± 0.2** |  | **0.1 ± 0.0** | **0.625** |  |  |  |
| **Height – m** | | **1.6 ± 0.1** |  | **1.6 ± 0.1** | **0.648** |  |  |  |
| **Weight – kg** | | **60.6 ± 11.9** |  | **52.5 ± 10.5** | **0.378** |  |  |  |
| **Body mass index –**  **kg/m^2^** | | **24.3 ± 2.9** |  | **20.7 ± 7.0** | **0.189** |  | | |
| **CHADS2** | | | | | | | | |
| **0, *n* (%)** | | **0 (0)** |  | **0 (0)** | **0.086** | **N.A.** | | |
| **1, *n* (%)** | | **1 (7.7)** |  | **1 (50.0)** |  |  |  |  |
| **2, *n* (%)** | | **8 (61.5)** |  | **1 (50.0)** |  |  |  |  |
| **3, *n* (%)** | | **2 (15.4)** |  | **0 (0)** |  |  |  |  |
| **4, *n* (%)** | | **2 (15.4)** |  | **0 (0)** |  |  |  |  |
| **5, *n* (%)** | | **0 (0)** |  | **0 (0)** |  |  |  |  |
| **Medication** | | | | | | | | |
| **ACEI/ARB, *n* (%)** | | **6 (46.2)** |  | **1 (50.0)** | **1.000** | **N.A.** | | |
| **Beta-blocker, *n* (%)** | | **9 (69.2)** |  | **1 (50.0)** | **1.000** |  |  |  |
| **Amiodarone, *n* (%)** | | **1 (7.7)** |  | **0 (0)** | **1.000** |  |  |  |
| **Antiarrhythmic, *n* (%)** | | **1 (7.7)** |  | **0 (0)** | **1.000** |  |  |  |
| **Echocardiographic parameter** | | | | | | | | |
| **Left atrial diameter –mm** | | **44.5 ± 4.5** |  | **39.7 ± 1.1** | **0.169** | **N.A.** | | |
| **Left ventricular ejection fraction – %** | | **61.3 ± 14.3** |  | **69.3 ± 20.5** | **0.493** |  |  |  |
| **E/e'** | | **12.9 ± 4.7** |  | **11.9 ± 3.7** | **0.765** |  |  |  |

Plus-minus values are means ± SD. IQR means interquartile range.
