# Supplementary material for: Predictors of outcome after catheter ablation for atrial fibrillation: Group analysis categorized by age and type of atrial fibrillation
Source: Ann Noninvasive Electrocardiol. 2022 Dec 16;28(2):e13020. doi: 10.1111/anec.13020 (PMC10023880; doi:10.1111/anec.13020)
Supplement: Supplementary file 4 — Table S3 [file ANEC-28-e13020-s005.docx]

**Table S3. Baseline and procedural characteristics of three groups, Younger PAF, Older PAF and PeAF.**

|  |  | **Younger, PAF** | | | **Older, PAF** | | | **PeAF** | | | **p value** |
| --- | --- | --- | --- | --- | --- | --- | --- | --- | --- | --- | --- |
|  |  | **(n=186)** | | | **(n=53)** | | | **(n=157)** | | |  |
| **Male sex (%)** | | **135** |  | **(72.6)** | **24** |  | **(45.3)** | **126** |  | **(80.3)** | **<0.001** |
| **Age (years)** | | **64.0** | **±** | **10.1** | **79.1** | **±** | **2.8** | **63.2** | **±** | **10.8** | **<0.001** |
| **Stroke (%)** | | **16** |  | **(8.6)** | **8** |  | **(15.1)** | **10** |  | **(6.4)** | **0.146** |
| **Hypertension (%)** | | **108** |  | **(58.1)** | **37** |  | **(70.0)** | **93** |  | **(59.2)** | **0.293** |
| **Diabetes (%)** | | **23** |  | **(12.4)** | **8** |  | **(15.1)** | **30** |  | **(19.1)** | **0.226** |
| **Laboratory data** | |  |  |  |  |  |  |  |  |  |  |
|  | **Creatinine (mg/dL)** | **0.9** | **±** | **0.2** | **0.9** | **±** | **0.3** | **1.0** | **±** | **0.6** | **0.043** |
|  | **Creatinine clearance (mL/min)** | **84.6** | **±** | **32.3** | **55.1** | **±** | **16.7** | **82.0** | **±** | **30.0** | **<0.001** |
|  | **NT-proBNP (pg/mL)** | **269.1** | **±** | **417.0** | **540.0** | **±** | **963.4** | **929.6** | **±** | **2395.0** | **<0.001** |
|  | **HbA1c (%)** | **5.8** | **±** | **1.0** | **5.9** | **±** | **0.5** | **6.0** | **±** | **0.6** | **0.247** |
|  | **C-reactive protein (mg/dL)** | **0.3** | **±** | **1.3** | **0.1** | **±** | **0.2** | **0.2** | **±** | **0.5** | **0.556** |
|  | **Height (m)** | **1.6** | **±** | **0.1** | **1.6** | **±** | **0.1** | **1.7** | **±** | **0.1** | **<0.001** |
|  | **Weight (kg)** | **66.9** | **±** | **13.0** | **58.8** | **±** | **10.8** | **70.2** | **±** | **14.4** | **<0.001** |
|  | **Body mass index (kg/m2)** | **24.6** | **±** | **3.9** | **24.0** | **±** | **3.4** | **25.4** | **±** | **4.4** | **0.060** |
| **CHADS2** | |  |  |  |  |  |  |  |  |  |  |
|  | **0** | **64** |  | **(34.4)** | **0** |  | **(0.0)** | **40** |  | **(25.5)** | **<0.0001** |
|  | **1** | **78** |  | **(41.9)** | **8** |  | **(15.1)** | **66** |  | **(42.0)** |  |
|  | **2** | **28** |  | **(15.1)** | **29** |  | **(54.7)** | **41** |  | **(26.1)** |  |
|  | **3** | **13** |  | **(7.0)** | **11** |  | **(20.8)** | **6** |  | **(3.8)** |  |
|  | **4** | **3** |  | **(1.6)** | **4** |  | **(7.5)** | **4** |  | **(2.6)** |  |
|  | **5** | **0** |  | **(0.0)** | **1** |  | **(1.9)** | **0** |  | **(0.0)** |  |
| **Medication at inhospitalization** | |  |  |  |  |  |  |  |  |  |  |
|  | **ACEI/ARB (%)** | **69** |  | **(37.1)** | **24** |  | **(45.3)** | **69** |  | **(44.0)** | **0.343** |
|  | **Beta-blocker (%)** | **78** |  | **(41.9)** | **21** |  | **(39.6)** | **75** |  | **(47.8)** | **0.441** |
|  | **Amiodarone (%)** | **16** |  | **(8.6)** | **2** |  | **(3.8)** | **33** |  | **(21.0)** | **0.0003** |
|  | **Antiarrhythmic**  **(%)** | **48** |  | **(25.8)** | **6** |  | **(11.3)** | **18** |  | **(11.5)** | **0.001** |
| **Measurements by echocardiogram** | |  |  |  |  |  |  |  |  |  |  |
|  | **Left atrial diameter (mm)** | **38.5** | **±** | **5.5** | **39.6** | **±** | **5.6** | **42.7** | **±** | **5.0** | **<0.0001** |
|  | **Left ventricular ejection fraction (%)** | **65.5** | **±** | **8.5** | **65.6** | **±** | **9.8** | **59.9** | **±** | **11.2** | **<0.0001** |
|  | **E/e'** | **11.0** | **±** | **4.3** | **14.8** | **±** | **6.0** | **10.7** | **±** | **3.7** | **<0.0001** |
