# Supplementary material for: Predictors of outcome after catheter ablation for atrial fibrillation: Group analysis categorized by age and type of atrial fibrillation
Source: Ann Noninvasive Electrocardiol. 2022 Dec 16;28(2):e13020. doi: 10.1111/anec.13020 (PMC10023880; doi:10.1111/anec.13020)
Supplement: Supplementary file 5 — Table S4 [file ANEC-28-e13020-s003.docx]

**Table S4. Predictor of AF recurrence in PeAF group**

|  |  |  |  |  |  | **Multivariate analysis** | | | |
| --- | --- | --- | --- | --- | --- | --- | --- | --- | --- |
|  |  | **AF recurrence (-)** |  | **AF recurrence (+)** | **p value** | **Odds ratio** | **95% CI** | **p value** | |
|  |  | **(n=123)** |  | **(n=34)** |  |  |  |  | |
| **Male sex (%)** | | **98 (79.7)** |  | **28 (82.4)** | **0.812** | **1.10** | **0.42-3.29** | **0.848** | |
| **Age (years, mean±SD)** | | **63.5 ± 10.9** |  | **62.1 ±10.5** | **0.502** | **1.01** | **0.97-1.04** | **0.711** | |
| **Stroke (%)** | | **8 (6.5)** |  | **2 (5.9)** | **1.000** |  |  |  | |
| **Hypertension (%)** | | **78 (63.4)** |  | **15 (44.1)** | **0.05** |  |  |  | |
| **Diabetes (%)** | | **25 (20.3)** |  | **5 (14.7)** | **0.623** |  |  |  | |
| **Creatinine (mg/dL)** | | **1.0 ± 0.7** |  | **1.0 ± 0.4** | **0.981** |  |  |  | |
| **Creatinine clearance (mL/min)** | | **82.1 ± 30.9** |  | **81.7 ± 27.0** | **0.950** |  |  |  | |
| **NT-proBNP (pg/mL)** | | **982.2 ± 216** |  | **739.3 ± 412** | **0.602** |  |  |  | |
| **HbA1c (%)** | | **6.0 ± 0.6** |  | **5.8 ± 0.5** | **0.235** |  |  |  | |
| **C-reactive protein (mg/dL)** | | **0.2 ± 0.5** |  | **0.2 ± 0.5** | **0.979** |  |  |  | |
| **Height (m)** | | **1.7 ± 0.1** |  | **1.7 ± 0.1** | **0.578** |  |  |  | |
| **Weight (kg)** | | **70.4 ± 15.0** |  | **69.4 ± 12.3** | **0.787** |  |  |  | |
| **Body mass index (kg/m2)** | | **25.5 ± 4.5** |  | **25.0 ± 3.8** | **0.534** |  |  |  | |
| **CHADS2** | | | | | | | | |  |
| **0** | | **28 (22.8)** |  | **12 (35.3)** | **0.219** |  |  |  | |
| **1** | | **50 (40.7)** |  | **16 (47.1)** |  |  |  |  | |
| **2** | | **36 (29.3)** |  | **5 (14.7)** |  |  |  |  | |
| **3** | | **6 (4.9)** |  | **0 (0.0)** |  |  |  |  | |
| **4** | | **3 (2.4)** |  | **1 (2.9)** |  |  |  |  | |
| **5** | | **0 (0.0)** |  | **0 (0.0)** |  |  |  |  | |
| **Medication** | | | | | | | | |  |
| **ACEI/ARB (%)** | | **59 (48.0)** |  | **10 (29.4)** | **0.078** | **0.46** | **0.195-1.030** | **0.059** | |
| **Beta-blocker (%)** | | **63 (51.2)** |  | **12 (35.3)** | **0.122** |  |  |  | |
| **Amiodarone (%)** | | **26 (21.1)** |  | **7 (20.6)** | **1.000** |  |  |  | |
| **Antiarrhythmic (%)** | | **13 (10.6)** |  | **5 (14.7)** | **0.545** |  |  |  | |
| **Echocardiographic parameter** | | | | | | | | |  |
| **Left atrial diameter (mm)** | | **42.5 ± 5.0** |  | **43.5 ± 4.6** | **0.277** |  |  |  | |
| **Left ventricular ejection fraction (%)** | | **59.1 ± 11.6** |  | **62.8 ±9.5** | **0.089** |  |  |  | |
| **E/e'** | | **10.7 ± 3.8** |  | **10.7 ± 3.4** | **0.931** |  |  |  | |
